# Targeting the epigenetic addiction of Merkel cell carcinoma

**DOI:** 10.15252/emmm.202013347

**Published:** 2020-10-16

**Authors:** Federico Mauri, Cédric Blanpain

**Affiliations:** ^1^ Laboratory of Stem Cells and Cancer Université Libre de Bruxelles (ULB) Brussels Belgium; ^2^ WELBIO Université Libre de Bruxelles (ULB) Bruxelles Belgium

**Keywords:** Cancer, Chromatin, Epigenetics, Genomics & Functional Genomics, Skin

## Abstract

Merkel cell carcinoma (MCC) is a rare but very aggressive neuroendocrine cancer of the skin, with very limited therapeutic options. Although immunotherapy is effective in some cases, there is an unmet need for new therapeutic approaches in MCCs. In this issue of *EMBO Molecular Medicine*, Leiendecker *et al* identify a selective vulnerability of MCC for inhibitors of the lysine‐specific histone demethylase 1A (LSD1). LSD1 inhibitors promote differentiation of tumor cells toward normal Merkel cell fate, impairing tumor cell growth *in vivo*, and opening new avenues for the treatment of patients with MCC.

## Graphical abstract

F. Mauri and C. Blanpain discuss the identification of a selective vulnerability of Merkel cell carcinomas for LSD1, which could be used for establishing new therapeutic strategies, as reported by A. Obenauf and colleagues in this issue of *EMBO Mol. Med*.

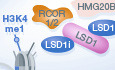

Merkel cells are neuroendocrine cells of the skin that are important for fine touch sensation (Lumpkin *et al*, 2010), arising from epidermal progenitors during embryonic development (Van Keymeulen *et al*, 2009). Their specification from epidermal cells relies on the activity of distinct pro‐neural transcription factors including Atoh1 and Sox2 (Maricich *et al*, 2009; Van Keymeulen *et al*, 2009; Bardot *et al*, 2013).

Merkel cell carcinoma (MCC), which is thought to arise from Merkel cells, is a relatively rare but very aggressive neuroendocrine cancer of the skin, associated with advanced age and immunosuppression. Although it represents less than 1% of all non‐melanoma skin cancers, its incidence has raised in the last years. Due to its very rapid metastatic spread, the mortality of MCC is very high (33–46% mortality rate) (Harms *et al*, 2018). In 80% of the cases, it is caused by the integration of the Merkel cell polyomavirus (MCV), which promotes tumorigenesis through the cooperation of large T viral antigen, that inactivates the Retinoblastoma tumor suppressor gene, and small T viral antigen that activates the MYCL‐EP400 transcriptional complex (Harms *et al*, 2018). Although immunotherapy is effective in a fraction of cases, the therapeutic options for the treatment of MCC are very limited (Nghiem *et al*, 2016).

Two recent studies, including one published in this issue of *EMBO Molecular Medicine* and the other recently published in *Nature Cell Biology*, identified LSD1 inhibition as a new therapeutic strategy to treat MCC (Leiendecker *et al*, 2020; Park *et al*, 2020). Using an *in vitro* drug screen with compounds targeting epigenetic regulators, Leiendecker *et al* identified an LSD1 inhibitor (LSD1i) as a specific and potent drug impairing the growth of MCV‐positive MCC cell lines *in vitro*, without affecting the growth of skin fibroblasts. LSD1 is a histone demethylase that mediates the removal of mono‐ and dimethylation marks on H3K4, which are linked to active transcription, thus promoting repression of gene expression (Shi *et al*, 2004). ShRNA knockdown of LSD1 impairs the growth of MCC cells *in vitro*, similarly to LSD1 pharmacological inhibition.

**Figure 1:**
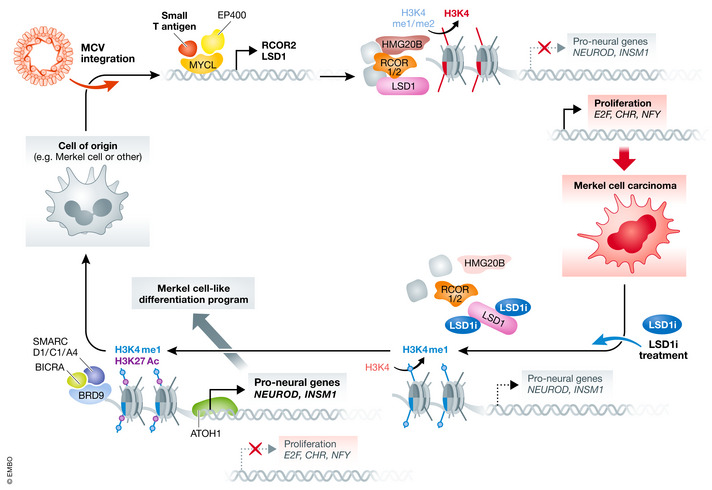
Inhibition of LSD1 impairs MCC transcriptional program After MCV integration, the small T antigen forms a complex with MYCL and EP400, driving the expression of LSD1 and other CoREST complex members. The LSD1‐CoREST complex mediates oncogenic transformation by repressing pro‐neural genes through direct removal of H3K4 mono‐/dimethylation marks, while indirectly promoting tumor cell proliferation. LSD1i treatment destabilizes the LSD1‐CoREST complex through the degradation of HMG20B. The resulting changes in Histone marks deposition allow the ncBAF complex to recognize the H3K27 acetylation marks through BRD9 and mediate the expression of pro‐differentiation genes, also with ATOH1 activity, restoring a Merkel cell‐like differentiation program.

To assess whether LSD1i was effective *in vivo*, the authors transplanted human MCC cell lines into immunodeficient mice and treated them with LSD1i. The treatment prevented tumor growth, whereas all control mice developed deadly tumors within 2 months. LSD1i also impaired the growth of established tumors. The treated mice did not develop severe side effects, demonstrating the efficacy and tolerability of LSD1i for MCC treatment *in vivo*.

To assess the cellular mechanism by which LSD1i impairs MCC growth, the authors assessed MCC proliferation and cell death following treatment. LSDi impaired MCC proliferation *in vitro* and *in vivo*, and induced MCC cell death by a caspase 3‐dependent and caspase 3‐independent mechanism.

To define the molecular mechanisms involved, Leiendecker *et al* performed a transcriptional profiling of MCC cells treated with LSD1i, and identified 870 upregulated and 533 downregulated genes compared to vehicle‐treated cells. The downregulated genes include many genes regulating cell cycle and DNA replication, whereas the upregulated genes show an enrichment for pathways involved in neuronal development and differentiation (Fig 1). Using SLAM‐seq, a method to identify newly transcribed genes, the authors demonstrated that pro‐neural genes such as NEUROD1 and INSM1 were upregulated almost immediately following LSD1i, as were components of the BMP signaling pathway. Interestingly, a similar set of genes and pathways upregulated by LSD1i were found by Park *et al*.

By comparing the genes upregulated by LSD1i in MCCs to the genes naturally expressed by normal Merkel cells, Leiendecker *et al* suggested that LSD1i promoted the differentiation of MCC into Merkel‐like cells. Consistent with this notion, using ChIP‐seq, Park *et al* found that LSD1 and its binding partners compete with ATOH1, a master regulator of Merkel cell fate, for binding to an overlapping set of genes, further supporting the idea that LSD1 competes for Atoh1 binding sites and represses the normal Merkel cell transcriptional program.

The two groups dissected in more detail the molecular mechanisms by which LSD1 sustains MCC tumor phenotype. By performing immunoprecipitation followed by mass spectrometry analysis, the authors of both groups showed that LSD1 interacts with members of the CoREST repressor complex including the core members HDAC1/2, the scaffolding proteins RCOR1‐3, and several non‐canonical components such as HMG20A/B. Leiendecker *et al* demonstrated that LSD1i leads to the loss of HMG20B binding, thereby impairing the assembly and stability of the LSD1‐CoREST complex. Supporting the notion that the loss of HMGB20 from this complex is important for the therapeutic effect of LSD1i, knockdown of HMGB20 also impaired MCC cell growth. Interestingly, a short pulse of LSD1i was sufficient to promote long‐term increase of pro‐neural gene expression and disassembly of the LSD1‐CoREST complex, leading to sustained pro‐neural differentiation and loss of tumorigenic potential.

Park *et al* performed a genome‐wide CRISPR screen to identify genes that would be positively or negatively selected upon LSD1i treatment. The level of histone lysine methylation marks is regulated in a coordinated manner by the activity of methyltransferases (“writers”) and demethylases (“erasers”, like LSD1). But the effect of such modifications depends on chromatin effector molecules (“readers”) that can recognize those marks and mediate chromatin remodeling and the associated transcriptional changes (Hyun *et al*, 2017). The screen of Park *et al* showed that several components of the non‐canonical BAF (ncBAF) chromatin‐remodeling complex (“reader”), including BRD9, were positively selected upon LSD1 inhibition, indicating that they are mediating the downstream effects of LSD1i. Park *et al* demonstrated that BRD9 and the ncBAF complex compete with LSD1 for binding to overlapping target genes, and that BRD9 mediates chromatin opening and the transcriptional activation of LSD1 targets after LSDi treatment.

In conclusion, these two studies elegantly demonstrate that pharmacological LSD1 inhibition is very efficient to promote the differentiation of MCCs into normal Merkel‐like cells strongly impairing MCC growth. These results show that in addition to leukemia, a subtype of colorectal cancer and basal cell carcinoma (Sanchez‐Danes *et al*, 2018; de The, 2018), therapies that induce differentiation could be very effective in the treatment of solid tumors. Epigenetic regulators targeting could be very efficient to achieve this goal, maximizing therapeutic benefits and minimizing side effects.

## References

[emmm202013347-bib-0001] Bardot ES , Valdes VJ , Zhang J , Perdigoto CN , Nicolis S , Hearn SA , Silva JM , Ezhkova E (2013) Polycomb subunits Ezh1 and Ezh2 regulate the Merkel cell differentiation program in skin stem cells. EMBO J 32: 1990–2000 23673358 10.1038/emboj.2013.110 PMC3715854

[emmm202013347-bib-0002] Harms PW , Harms KL , Moore PS , DeCaprio JA , Nghiem P , Wong MKK , Brownell I , International Workshop on Merkel Cell Carcinoma Research Working Group (2018) The biology and treatment of Merkel cell carcinoma: current understanding and research priorities. Nat Rev Clin Oncol 15: 763–776 30287935 10.1038/s41571-018-0103-2 PMC6319370

[emmm202013347-bib-0003] Hyun K , Jeon J , Park K , Kim J (2017) Writing, erasing and reading histone lysine methylations. Exp Mol Med 49: e324 28450737 10.1038/emm.2017.11 PMC6130214

[emmm202013347-bib-0004] Leiendecker L , Jung PS , Krecioch I , Neumann T , Schleiffer A , Mechtler K , Wiesner T , Obenauf AC (2020) LSD1 inhibition induces differentiation and cell death in Merkel cell carcinoma. EMBO Mol Med 12: e12525 10.15252/emmm.202012525 PMC7645387 33026191

[emmm202013347-bib-0005] Lumpkin EA , Marshall KL , Nelson AM (2010) The cell biology of touch. J Cell Biol 191: 237–248 20956378 10.1083/jcb.201006074 PMC2958478

[emmm202013347-bib-0006] Maricich SM , Wellnitz SA , Nelson AM , Lesniak DR , Gerling GJ , Lumpkin EA , Zoghbi HY (2009) Merkel cells are essential for light‐touch responses. Science 324: 1580–1582 19541997 10.1126/science.1172890 PMC2743005

[emmm202013347-bib-0007] Nghiem PT , Bhatia S , Lipson EJ , Kudchadkar RR , Miller NJ , Annamalai L , Berry S , Chartash EK , Daud A , Fling SP *et al* (2016) PD‐1 Blockade with Pembrolizumab in Advanced Merkel‐Cell Carcinoma. N Engl J Med 374: 2542–2552 27093365 10.1056/NEJMoa1603702 PMC4927341

[emmm202013347-bib-0008] Park DE , Cheng J , McGrath JP , Lim MY , Cushman C , Swanson SK , Tillgren ML , Paulo JA , Gokhale PC , Florens L *et al* (2020) Merkel cell polyomavirus activates LSD1‐mediated blockade of non‐canonical BAF to regulate transformation and tumorigenesis. Nat Cell Biol 22: 603–615 32284543 10.1038/s41556-020-0503-2 PMC7336275

[emmm202013347-bib-0009] Sanchez‐Danes A , Larsimont JC , Liagre M , Munoz‐Couselo E , Lapouge G , Brisebarre A , Dubois C , Suppa M , Sukumaran V , Del Marmol V *et al* (2018) A slow‐cycling LGR5 tumour population mediates basal cell carcinoma relapse after therapy. Nature 562: 434–438 30297799 10.1038/s41586-018-0603-3 PMC6295195

[emmm202013347-bib-0010] Shi Y , Lan F , Matson C , Mulligan P , Whetstine JR , Cole PA , Casero RA , Shi Y (2004) Histone demethylation mediated by the nuclear amine oxidase homolog LSD1. Cell 119: 941–953 15620353 10.1016/j.cell.2004.12.012

[emmm202013347-bib-0011] de The H (2018) Differentiation therapy revisited. Nat Rev Cancer 18: 117–127 29192213 10.1038/nrc.2017.103

[emmm202013347-bib-0012] Van Keymeulen A , Mascre G , Youseff KK , Harel I , Michaux C , De Geest N , Szpalski C , Achouri Y , Bloch W , Hassan BA *et al* (2009) Epidermal progenitors give rise to Merkel cells during embryonic development and adult homeostasis. J Cell Biol 187: 91–100 19786578 10.1083/jcb.200907080 PMC2762088

